# Effects of inoculation and dry matter content on microbiome dynamics and metabolome profiling of sorghum silage

**DOI:** 10.1007/s00253-024-13096-4

**Published:** 2024-03-08

**Authors:** Zohreh Akhavan Kharazian, Dongmei Xu, Rina Su, Xusheng Guo

**Affiliations:** 1 State Key Laboratory of Grassland and Agro-Ecosystems, School of Life Sciences, Lanzhou University, Lanzhou, 730000 China; 2 Probiotics and Biological Feed Research Center, Lanzhou University, Lanzhou, 730000 China

**Keywords:** Sorghum silage, Lactic acid bacteria, Microbial community, Bio-functional metabolite

## Abstract

**Abstract:**

Sorghum forage was ensiled for 90 days at two dry matter (DM) contents (27 vs. 39%) without or with *Lactiplantibacillus plantarum* inoculation. On day 90 of fermentation, silages were sampled to assess the microbial community dynamics and metabolome profile. *L. plantarum* inoculation improved silage quality, as shown by a lower pH and greater acetic acid concentration. Loss of DM remained unaffected by *L. plantarum* inoculation but was greater in low- vs. high-DM sorghum silages (14.4 vs. 6.62%). The microbiome analysis revealed that *Pseudomonas congelans* represented the dominant species of the epiphytic microbiota in both low- and high-DM sorghum forage before ensiling. However, *L. buchneri* represented the dominant species at the end of ensiling. Ensiling fermentation resulted in distinct metabolic changes in silages with varying DM content. In low-DM silages, ensiling fermentation led to the accumulation of 24 metabolites and a reduction in the relative concentration of 13 metabolites. In high-DM silages, ensiling fermentation resulted in an increase in the relative concentration of 26 metabolites but a decrease in the concentration of 8 metabolites. Compared to non-inoculated silages, *L. plantarum* inoculation resulted in an increased concentration of 3 metabolites and a reduced concentration of 5 metabolites in low-DM silages. Similarly, in high-DM silages, there was an elevation in the relative concentration of 3 metabolites, while a decrease in 7 other metabolites. Ten metabolites with bio-functional activity were identified, including chrysoeriol, isorhamnetin, petunidin 3-glucoside, apigenin, caffeic acid, gallic acid, *p*-coumaric acid, trans-cinnamic acid, herniarin, and 3,4-dihydroxy-trans-cinnamate. This study presents a comprehensive analysis of microbiome and metabolome profiling of sorghum forage during ensiling as a function of DM content and *L. plantarum* inoculation, with a particular emphasis on identifying metabolites that may possess bio-functional properties.

**Key points:**

• *DM loss was not different by L. plantarum but higher in low- vs. high-DM silage.*

• *L. buchneri dominated ensiling, regardless of DM level.*

• *10 metabolites with bio-functional activity were identified.*

## Introduction

Ensiling is a simple and widespread method of fresh forage conservation for long-term use enabled by lactic acid fermentation using epiphytic lactic acid bacteria (LAB) under anaerobiosis (McDonald et al. 1991). Conversion of soluble sugars into organic acids occurs using LAB, causing silage pH to decline, thereby contributing to forage preservation if exposure to air is limited (McDonald et al. 1991). Sorghum forage is extensively grown in Southern China for ruminant forage production purposes. The carbohydrate content in sorghum is similar to sugarcane and corn forage, but it has substantially lower water and fertilizer requirements. In addition, flexible planting time, rapid growth, high nutritive value, high resistance to temperatures, and high biomass yield make sorghum an attractive plant for cultivation in Southern China (Almodares et al. 2011; Chen et al. 2019; Diepersloot et al. 2021). In the eastern regions of China, sweet sorghum has demonstrated better resilience to elevated temperatures, excessive water, clay soil, and acidic pH levels compared to corn (Qu et al. 2014).

Epiphytic LAB naturally initiates multiplying and acidifying forage biomass. However, homofermentative LAB inoculant strains have been added at ensiling to accelerate forage biomass acidification, in order to minimize nutrient loss during the ensiling process and thus improve the silage quality by preventing the proliferation of spoilage microorganisms (Ranjit and Kung 2000; Liu et al. 2014; Muck et al. 2018). However, the efficacy of LAB inoculants in improving the silage fermentation quality is crop-specific (Oliveira et al. 2017), necessitating additional experiments to clarify the effects of the LAB inoculants on the silage fermentative patterns.

Dry matter (DM) content at ensiling is a critical factor affecting the quality of silage fermentation, feed intake, and animal productivity (Guo et al. 2013). Inappropriate moisture content may limit the fermentative process during ensiling (Hu et al. 2009). Thus, achieving efficient silage fermentation may necessitate ensuring appropriate DM content and LAB inoculants.

The process of silage fermentation is dynamic, characterized by shifts in microbial communities and alterations in metabolites as fermentation progresses. Understanding the contribution of microbial communities in silage production is critical for identifying the appropriate microorganisms and controlling the proliferation of harmful pathogens (Driehuis 2013). However, the traditional enumeration methods may not accurately identify the silage microbial community, as some microorganisms cannot be cultured using traditional techniques (Guan et al. 2020). The increasing use of next-generation sequencing enables the identification and estimation of the effects of various physical or biological factors, such as crop or inoculant type and DM content, on the microbiome dynamics in silage samples (McDonald et al. 1991; McAllister et al. 2018).

Metabolomics technology has provided a vast opportunity to comprehensively identify alterations in metabolites in response to disturbance or stimulation, and recently, metabolomic profiling analysis is being extensively used in silage research, holding great potential to identify novel metabolites produced from the complex biochemical reactions occurring during ensiling (Guo et al. 2018). This may allow for a clearer understanding of how metabolites change in response to different conditions, such as inoculation or moisture level at ensiling (Guo et al. 2018; Xu et al. 2019).

Sorghum is globally grown and is a rich source of phenolic compounds. Phenolic compounds are plant secondary metabolites, and they may positively affect animal performance, stress mitigation, gut microflora development, and immune function (Formato et al. 2022). Moreover, phenolic compounds show anti-inflammatory, antioxidant, antimicrobial, antimutagenic, and immunomodulatory properties in many organisms (Waqas et al. 2023). Phenolic compounds, acquired from plants as a component of the cattle diet, may potentially play a vital role in animal health (Tsen et al. 2014), and identification of these phenolic compounds would provide invaluable information about silage nutritional quality relevant to animal health (Xu et al. 2020, 2021).

There is limited information about how DM content and *L. plantarum* (LP) inoculation at ensiling interact and affect the quality of silage fermentation, bacterial community, and metabolome changes, including phenolic compounds in whole-plant sorghum silage. We theorized that the DM content at ensiling may affect the efficacy of LP inoculant on the silage fermentation quality of sorghum forage. Therefore, this study aimed to assess the quality of fermentative silage, bacterial communities, and metabolome profiling of whole-plant sorghum that was ensiled at two DM contents with or without LP inoculation.

## Materials and methods

Sweet sorghum variety (DAKA) was grown in Gansu Province, Tongwei County (Dingxi city). A fodder chopper was used to chop the sorghum plant into 2 cm cuts after 12 weeks of growth. Harvested sorghum forage with an initial DM content of approximately 27% was immediately transported to the laboratory and wilted [temperature = 28–30°C; relative humidity = 70%] for 5 h to reach the DM concentration of 39%. Chopped sorghum forage was sprayed with *L. plantarum* MTD/1 inoculant (LP, Crop-N-Rich®500, Vita Plus, Madison, WI, USA) at 1 × 10^6^ cfu/g fresh weight. The inoculant was suspended in sterile water to achieve a 10 mL/kg fresh weight ratio. Low- and high-DM sorghum forage with or without LP inoculation was ensiled. Chopped sorghum forage without inoculant was sprayed with an equal volume of distilled water. Upon mixing, approximately 200 g chopped sorghum mass (3 replications/treatment) was packed into vacuum-sealed polyethylene plastic bags (25 cm × 35 cm) and maintained for 90 days at room temperature (22–25°C).

### Silage quality characterization

Dry matter content was determined using a forced-air oven (65°C; 72 h). A 20-g sample was placed in a juice extractor, diluted with 180 mL of distilled water, and subjected to high-speed squeezing for 30 s. The suspension was filtered through four layers of gauze. The pH was determined using a glass electrode pH meter. Following the pH measurement, one portion of the filtrate was acidified using 7.14 M H_2_SO_4_ and filtered through a 0.45-μm dialyzer. The quantification of propionic acid, lactic acid, butyric acid, and acetic acid was performed using HPLC with a KC-811 column manufactured by Shodex (Shimadzu, Kyoto, Japan). The HPLC system was operated at a 1 mL/min flow rate and an oven temperature of 50°C. Detection was conducted at a wavelength of 210 nm using an SPD detector (Ke et al. 2022).

### Bacterial community composition

Analysis of bacterial community composition was described before (Xu et al. 2019). In brief, the full-length 16S rRNA was amplified for single-molecule real-time (SMRT) sequencing during DNA extraction from fresh and silage samples. The amplification was achieved using the forward primer 27F (5′-AGAGTTTGATCC TGGCTCAG-3′) and the reverse primer 1492R (5′-GGTTACCTTGTTACGA CTT-3′). Both amplification primers were equipped with a set of 16-nucleotide barcodes. The PCR program employed for amplification was as follows: an initial denaturation step at 95°C for 3 min, followed by 25 cycles consisting of denaturation at 98°C for 20 s, annealing at 57°C for 30 s, and extension at 72°C for 90 s. Finally, an extension step was performed at 72°C for 2 min. Quality control measures (Mosher et al. 2013) were implemented to ensure the reliability and accuracy of the PCR amplifications and preprocessing of sequences.

The PacBio Sequel instrument was used for sequencing the amplicons. The 16S rRNA library was created with a Pacific Biosciences template preparation kit. The raw data obtained from the sequencing process underwent extraction and filtering steps, and alpha diversity was determined (Yan et al. 2019a). To achieve accurate species annotations, we classified curated PacBio sequences using VSEARCH v2.14.1 with the –usearchglobal option and specific settings: –id 0.75 –blast6out –strand both –maxaccepts 5. We then combined these sequences with the last common ancestor (LCA) algorithm for improved reliability (Wood and Salzberg 2014). After the taxonomic annotation, non-bacterial taxa, including chloroplasts and mitochondria, were excluded from the analysis to prevent any interference with the results. The unweighted pair-group method subjected the cluster analysis to arithmetic average (UPGMA) and phylogeny-based (UniFrac) unweighted distances using QIIME.

### Comprehensive metabolomic profiling by LC–ESI–MS/MS

Using liquid chromatography-electrospray ionization-tandem mass spectrometry, we conducted the metabolomic analysis with HPLC separation on a Shim-pack UFLC SHIMADZU CBM30A system. The Applied Biosystems 4500 Q TRAP instrument was used to perform mass spectrometry, following the methodology described by Yan et al. (2019a). The analytical conditions for the HPLC analysis involved using a Waters ACQUITY UPLC HSS T3 C18 column (1.8 µm, 2.1 mm × 100 mm) for ultra-high-performance liquid chromatography separation. The mobile phase used was a combination of solvent A (0.1% formic acid) and solvent B (acetonitrile). The sample measurements were conducted using a gradient program, starting with the initial conditions of 95% solvent A and 5% solvent B. A linear gradient was programmed, gradually transitioning to 5% solvent A and 95% solvent B. The composition of the mobile phase was adjusted to 5% solvent A and 95% solvent B and maintained for 1 min. The composition was swiftly adjusted to 95% solvent A and 5% solvent B within 0.1 min and held for 2.9 min. The column oven temperature was set at 40°C, and the injection volume for each sample was 2 μL. The effluent from the HPLC system was alternately directed to the electrospray ionization (ESI)-triple quadrupole instrument. A detailed description of the qualitative and quantitative analysis of the metabolite and the preprocessing of raw data were reported before (Yan et al. 2019a). The high-resolution MS data were converted from its original format to the mzXML format using ProteoWizard. Subsequently, the processed data was analyzed using MAPS software (version 1.0). The preprocessing of the MS data resulted in the generation of a data matrix. This matrix contained information such as the retention time, mass-to-charge ratio (m/z) values, and peak intensity for each detected peak in the analyzed samples. Metabolite identification was performed using an in-house MS2 database. Additionally, the MRM (Multiple Reaction Monitoring) data obtained from the analysis were processed using Skyline software. To validate the putative metabolites with differential expression, two online databases were utilized: Human Metabolome Database (HMDB; http://www.hmdb.ca) and Kyoto Encyclopedia of Genes and Genomes (KEGG; http://www.genome.jp/kegg) and the Progenesis QI software was employed for this purpose. To evaluate metabolome alteration, we used the untargeted metabolomic approach. The cut-off for annotation was set at MS2 ≥ 0.3, and metabolite identification was reliable if the MS2 score was greater than 0.8 (Liu et al. 2022).

### Statistical analysis

The data in Table 1 and 2 were analyzed in a 2 × 2 factorially arranged design with main factors as LP inoculation and DM content at ensiling. The statistical analysis was performed in SAS (SAS 9.4, SAS Institute Inc., Cary, NC, USA). The treatment means differences were separated using Tukey’s test.

**Table 1:** Fermentation quality of sorghum silage as a function of dry matter (DM) content (27 vs. 39%) and *L. plantarum* application after 90 days of fermentation

Items	Treatments*				SEM	*P* value		
	Non-inoculated		LP-inoculated					
	Low-DM	High-DM	Low-DM	High-DM		DM	I	DM × I
Dry matter, %	24.7	39.1	22.7	38.7	2.23	< 0.01	0.13	0.30
Dry matter loss	12.7	5.93	20.4	7.30	1.93	< 0.01	0.11	0.24
pH	3.75	3.88	3.64	3.84	0.09	< 0.01	< 0.01	0.14
Lactic acid, g/kg DM	75.8	43.8	60.8	46.5	1.55	< 0.01	0.29	0.14
Acetic acid, g/kg DM	25.9	13.0	28.1	19.1	0.63	< 0.01	< 0.01	0.01
Propionic acid, g/kg DM	13.8	8.45	5.62	11.6	0.34	0.63	< 0.01	< 0.01

*Non-inoculated, silage without inoculation; *LP*, *L. plantarum* inoculation; *SEM*, standard error of the mean; *DM*, dry matter content effect; *I*, inoculant effect; *DM* × *I*, the interaction between DM content and inoculant

**Table 2:** Estimates of α-diversity in sorghum silages as a function of dry matter (DM) content (27 vs. 39%) and *L. plantarum* application after 90 days of fermentation

*α*-diversity	Treatments*				SEM	*P* value		
	Non-inoculated		LP-inoculated					
	Low-DM	High-DM	Low-DM	High-DM		DM	I	DM × I
OTU	30.0	39.3	15.0	34.0	6.37	0.01	0.04	0.29
Chao1	37.5	55.2	21.2	46.9	6.53	0.03	0.19	0.65
Simpson	0.96	0.74	0.98	0.92	0.06	0.03	0.09	0.17
Shannon	0.14	0.62	0.07	0.24	0.16	0.01	0.05	0.15
Coverage	0.999	0.999	0.999	0.999	–	0.20	0.31	0.46

*Non-inoculated, silage without inoculation; *LP*, *L. plantarum* inoculation; *SEM*, standard error of the mean; *OUT*, operational taxonomic unit; *DM*, dry matter content effect; *I*, inoculant effect; *DM* × *I*, the interaction between DM content and inoculant

## Results

### Silage fermentation quality

Silage pH, organic acid composition, and DM loss after 90-day ensiling are presented in Table 1. Butyric acid was undetectable in any of the samples assayed. Dry matter loss was greater in low- vs. high-DM silages. Silage pH was lower, but lactic acid and acetic acid were greater in low- vs. high-DM sorghum silages. Propionic acid remained unaffected with DM content. Silage pH and propionic acid were lower but acetic acid concentration was greater in LP-inoculated silages. Dry matter loss and lactic acid concentration remained affected with LP inoculation. Interaction effects between DM content and LP inoculation existed for propionic and acetic acid, as LP inoculation resulted in greater propionic acid concentration in high-DM silages (*P* < 0.01) but lower greater acetic acid concentration in low-DM silages (*P* = 0.01).

### Microbial diversity

Estimates of α-diversity in sorghum silage as a function of DM content and LP inoculation in 90-day silages are presented in Table 2. All samples showed an adequate coverage (> 0.99), implying the proper identification of most microbial communities. Irrespective of LP inoculation, Chao1 increased in high- vs. low-DM silages. Microbial communities during ensiling were distinctly different in silages with or without LAB inoculation, with Shannon index as a measure of bacterial diversity declining with LP inoculation compared with non-inoculated silages.

### Dynamic changes in bacterial communities

**Fig. 1:**
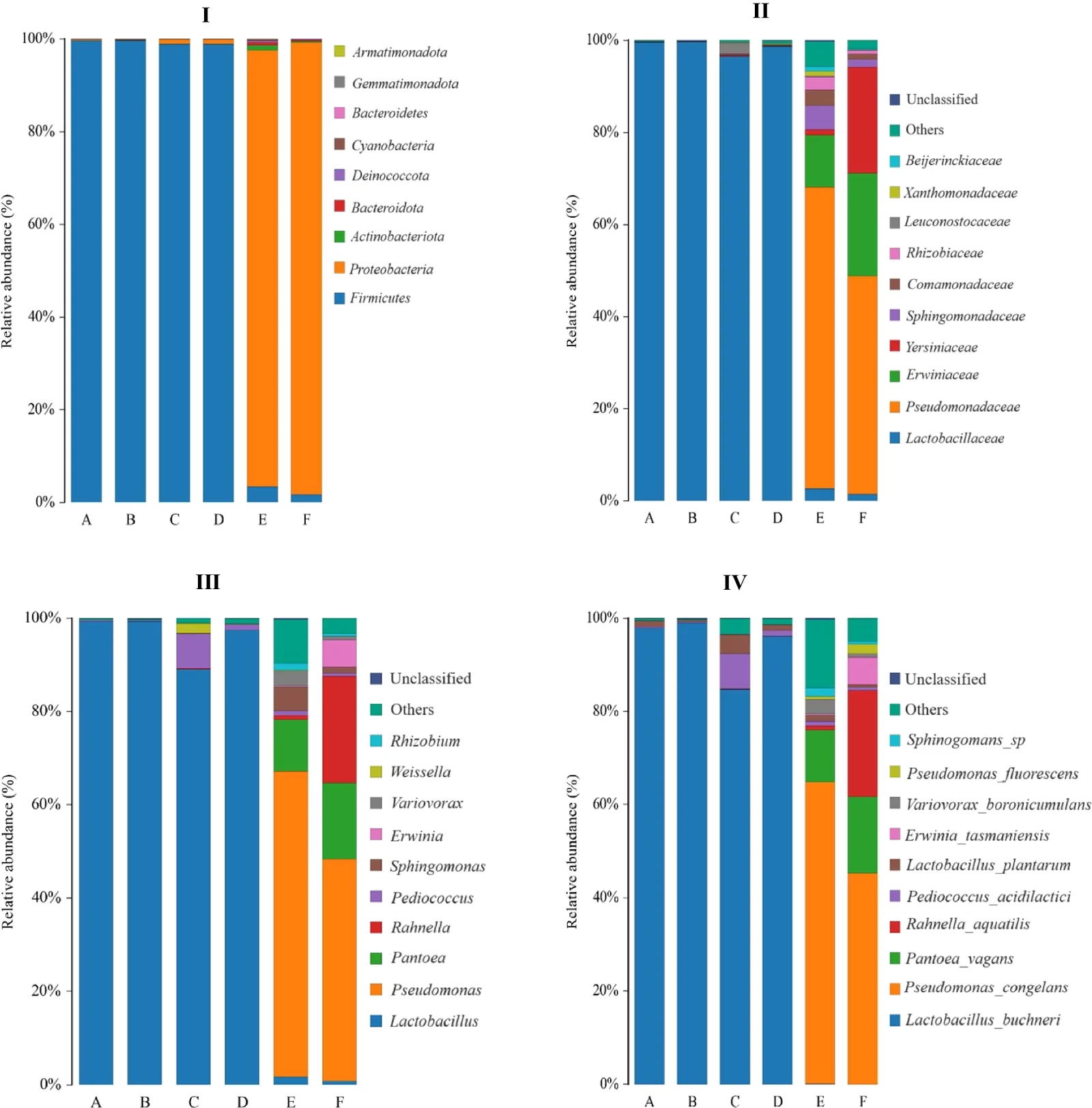
Relative abundance (%) of phyla (I), families (II), genera (III), and species (IV) isolated from low- (27%) vs. high-dry matter (DM; 39%) sorghum forage or ensiled with or without *L. plantarum* inoculation after 90 days of fermentation. A = low-DM silage without *L. plantarum* inoculation. B = low-DM silage with *L. plantarum* inoculation. C = high-DM silage without *L. plantarum* inoculation. D = high-DM silage with *L. plantarum* inoculation. E = low-DM sorghum forage before ensiling. F = high-DM sorghum forage before ensiling

The relative abundance of phylum, family, genus, and species as a function of DM content and LP inoculation in 90-day sorghum silages is presented in Fig. 1. At the phylum level, *Proteobacteria* represented the dominant phylum of the epiphytic microbiota in sorghum biomass before ensiling, regardless of DM content. However, at the end of 90-day ensiling, they were replaced by *Firmicutes*. *Pseudomonadaceae* represented the dominant family of the epiphytic microbiota in high-DM sorghum forage before ensiling, followed by *Erwiniaceae*. However, at the end of the ensiling, they were replaced by *Lactobacillaceae*.

In low-DM sorghum forage before ensiling, *Pseudomonadaceae* represented the dominant family of the epiphytic microbiota, followed by *Yersiniaceae.* Regardless of DM content at ensiling*, Lactobacillaceae* represented the dominant family of the epiphytic microbiota in 90-day silages. A similar pattern as the family level was observed at the genus level, with *Pseudomonas* representing the dominant genus of the epiphytic microbiota in high-DM sorghum forage before ensiling, followed by *Rahnella*, *Pantoea*, and then *Erwinia*. However, on day 90 of ensiling, they were mainly replaced by *Lactobacillus*. At the species level, *Pseudomonas congelans* represented the dominant species of the epiphytic microbiota in both low- and high-DM sorghum forage before ensiling, but *L. buchneri* replaced them at the end of ensiling. *Erwinia tasmaniensis* was the most abundant species after *L. buchneri* in high-DM silage without LP inoculation.

**Fig. 2:**
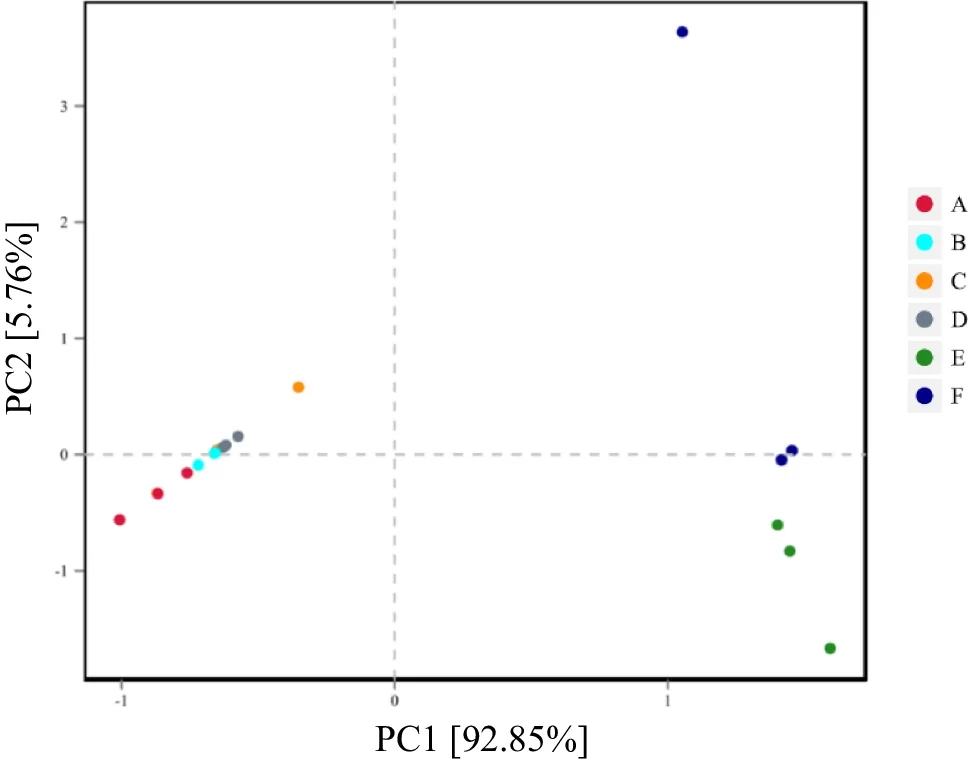
Principal component analysis (PCA) based on PC1 and PC2 showing variation in bacterial community structure. Each point represents an individual. A = low-DM silage without *L. plantarum* inoculation. B = low-DM silage with *L. plantarum* inoculation. C = high-DM silage without *L. plantarum* inoculation. D = high-DM silage with *L. plantarum* inoculation. E = low-DM sorghum forage before ensiling. F = high-DM sorghum forage before ensiling

Principal component analysis (PCA) illustrating variation in bacterial community structure is shown in Fig. 2. Regardless of DM content and LP inoculation, variation in microbial diversity was lower in 90-d silage as compared with fresh sorghum forage.

### Metabolome profiling

**Fig. 3:**
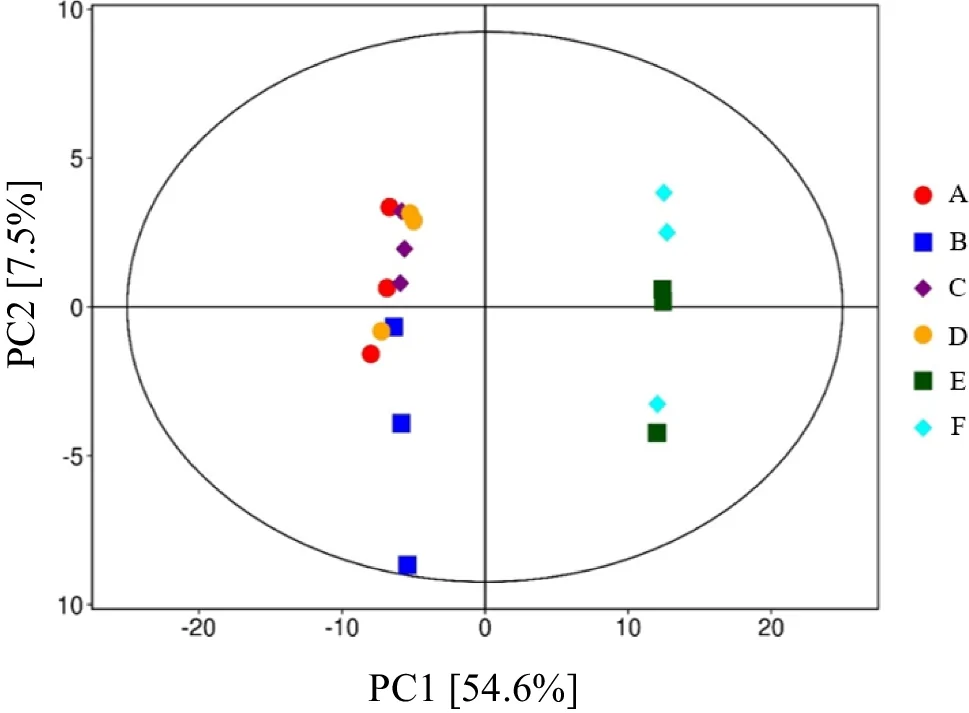
Principal component analysis (PCA) based on PC1 and PC2 showing variation in metabolic profiles in low- (27%) or high-dry matter (DM; 39%) sorghum silages without or with *L. plantarum* (LP) inoculation after 90 d of ensiling. A = low-DM silage without *L. plantarum* inoculation, B = low-DM silage with *L. plantarum* inoculation, C = high-DM silage without *L. plantarum* inoculation, D = high-DM silage with *L. plantarum* inoculation, E = low-DM sorghum forage before ensiling, F = high-DM sorghum forage before ensiling

The PCA plot of the metabolome profile of fresh or ensiled sorghum forage at two DM contents without or with LP inoculation is presented in Fig. 3, showing that regardless of DM content and LP inoculation, the ensiling fermentation resulted in clear separation from fresh biomass.

Tables 3 and 4 present the differential metabolites identified in low- and high-DM silages, respectively. After filtering metabolites with a score above 0.8, only 44 out of the 408 identified metabolites were retained. Compared to non-inoculated silages, there were relatively fewer mini peptides in both low- and high-DM silages treated with LP. In low-DM silages, LP inoculation resulted in an increased relative concentration of diphenylamine, gentisic acid, and herniarin, while a decrease in gamma-glutamyl leucine, Ile Gly, Ile Leu, Ser Gly Ile, and nicotinic acid as compared to non-inoculated silages. In high-DM silages, LP inoculation increased the relative concentration of gentisic acid, ornithine, and (A ±)-tryptophan, while causing a decrease in chrysoeriol, beta-carboline, Ile Gly, Ser Gly Ile, cytarabine, malic acid, and thymidine as compared to non-inoculated silages.

**Table 3:** Differential metabolites of low-dry matter (27%) sorghum silage with or without inoculation

Metabolites	Class	Relative concentration			Fold change		
		FM	CON	LP	Log_2_ (CON/FM)	Log_2_ (LP/CON)	Log_2_ (LP/FM)
2-Hydroxybenzaldehyde	Benzene and substituted derivatives	3.6E-03	9.7E-03	8.0E-03	1.43*	− 0.28	1.15
Dibutyl phthalate	Benzene and substituted derivatives	7.9E-04	2.8E-04	5.8E-04	− 1.50*	1.05	− 0.45
Diphenylamine	Benzene and substituted derivatives	9.9E-05	8.6E-05	1.5E-04	− 0.20	0.80*	0.60
Gallic acid	Benzene and substituted derivatives	2.4E-07	7.8E-06	6.5E-06	5.02*	− 0.26	4.76*
Gentisic acid	Benzene and substituted derivatives	1.4E-06	6.2E-05	2.0E-04	5.47*	1.69*	7.16*
Cis-aconitic acid	Carboxylic acids and derivatives	1.5E-02	9.7E-03	1.3E-02	− 0.63*	0.42	− 0.21
Gamma-glutamyl-leucine	Carboxylic acids and derivatives	2.9E-03	2.2E-04	7.1E-05	− 3.72*	− 1.63*	− 5.35*
L-Histidine	Carboxylic acids and derivatives	1.2E-04	3.8E-04	3.2E-04	1.66*	− 0.25	1.42
L-Methionine	Carboxylic acids and derivatives	3.6E-04	1.9E-02	1.3E-02	5.72*	− 0.55	5.17
L-Tyrosine	Carboxylic acids and derivatives	1.8E-04	3.7E-04	4.1E-04	1.04*	0.15	1.19
L-Valine	Carboxylic acids and derivatives	5.5E-05	6.7E-04	7.4E-04	3.61	0.14	3.75*
Ornithine	Carboxylic acids and derivatives	2.1E-03	1.6E-02	1.5E-02	2.93*	− 0.09	2.84*
Pipecolic acid	Carboxylic acids and derivatives	1.6E-03	3.3E-02	2.9E-02	4.37*	− 0.19	4.18*
Succinic acid	Carboxylic acids and derivatives	3.8E-05	8.5E-04	7.5E-04	4.48*	− 0.18	4.30*
Trans-aconitic acid	Carboxylic acids and derivatives	2.5E-04	1.5E-03	2.0E-03	2.58*	0.42	3.00*
3,4-Dihydroxy-trans-cinnamate	Cinnamic acids and derivatives	9.5E-06	1.4E-04	1.6E-04	3.88*	0.19	4.07
Coumarin	Coumarins and derivatives	3.6E-03	8.5E-03	1.2E-02	1.24*	0.50	1.74*

Cytosine	Diazines	1.5E-05	6.5E-06	1.3E-05	− 1.21*	1.00	− 0.21*
Citramalic acid	Fatty acyls	1.1E-05	1.6E-05	1.8E-05	0.54*	0.17	0.71
Apigenin	Flavonoids	1.7E-04	1.3E-03	1.2E-03	2.93	− 0.12	2.82*
Chrysoeriol	Flavonoids	2.2E-04	3.8E-03	3.5E-03	4.11*	− 0.12	3.99*
Isorhamnetin	Flavonoids	5.6E-05	4.2E-04	3.5E-04	2.91*	− 0.26	2.64*
Malvidin 3-glucoside	Flavonoids	2.7E-02	3.3E-03	5.4E-03	− 3.03	0.71	− 2.32*
Petunidin 3-glucoside	Flavonoids	1.6E-03	4.5E-04	6.0E-04	− 1.83*	0.42	− 1.42*
Malic acid	Hydroxy acids and derivatives	3.2E-03	9.5E-05	3.0E-04	− 5.07*	1.66	− 3.42*
Adenine	Imidazopyrimidines	1.1E-02	6.6E-05	8.4E-04	− 7.38*	3.67	− 3.71*
Uric acid	Imidazopyrimidines	4.7E-06	6.6E-05	7.6E-05	3.81*	0.20	4.02*
(Â ±)-Tryptophan	Indoles and derivatives	2.7E-02	3.3E-02	2.5E-02	0.29*	− 0.40	− 0.11
1H-Indole-3-carboxaldehyde	Indoles and derivatives	1.8E-05	7.3E-06	1.5E-05	− 1.30*	1.04	− 0.26
Beta-Carboline	Indoles and derivatives	2.3E-05	5.2E-04	8.9E-04	4.50*	0.78	5.27
Ile Gly	Mini peptide	3.8E-04	2.5E-03	5.0E-04	2.72*	− 2.32*	0.40
Ile Leu	Mini peptide	1.4E-03	1.2E-03	3.8E-04	− 0.22	− 1.66*	− 1.88*
Ser Gly Ile	Mini peptide	1.0E-04	2.3E-04	7.8E-05	1.20	− 1.56*	− 0.36
Ser Val Glu	Mini peptide	8.1E-04	1.0E-04	7.0E-05	− 3.02*	− 0.51	− 3.53*
Acetylcholine	Organonitrogen compounds	1.5E-04	4.6E-02	4.9E-02	8.26*	0.09	8.35*
Adenosine	Purine nucleosides	1.8E-01	1.1E-03	1.3E-02	− 7.35*	3.56	− 3.79*
Nicotinic acid	Pyridines and derivatives	2.2E-03	2.2E-03	1.0E-03	− 0.00	− 1.14*	− 1.14*
Pyridoxine	Pyridines and derivatives	3.0E-04	2.2E-03	1.8E-03	2.87*	− 0.29	2.58
Cytarabine	Pyrimidine nucleosides	1.1E-02	1.1E-04	7.4E-04	− 6.64*	2.75	− 3.89*
Thymidine	Pyrimidine nucleosides	4.0E-04	1.5E-05	4.4E-05	− 4.74*	1.55	− 3.18*
*p*-coumaric acid	Hydroxycinnamic acids	2.3E-05	8.8E-06	1.4E-05	− 1.40*	0.67	− 0.73
Caffeic acid	Hydroxycinnamic acids	3.5E-06	1.1E-03	8.8E-04	8.31*	− 0.34	7.97
Herniarin	Coumarins and derivatives	2.8E-03	6.2E-03	1.1E-02	1.17*	0.87*	2.04*
Trans-cinnamic acid	Cinnamic acids and derivatives	2.6E-03	8.5E-03	5.4E-03	1.72*	− 0.66	1.07

*FM*, fresh sorghum forage before ensiling; *CON*, sorghum silage without *L. plantarum* inoculation (control); *LP*, sorghum silage with *L. plantarum* inoculation

* Indication of a significant effect (*P* < 0.05)

**Table 4:** Differential metabolites of high-dry matter (39%) sorghum silage with or without inoculation

Metabolites	Class	Relative concentration			Fold change		
		FM	CON	LP	Log_2_ (CON/FM)	Log_2_ (LP/CON)	Log_2_ (LP/FM)
2-Hydroxybenzaldehyde	Benzene and substituted derivatives	2.6E-03	9.9E-03	1.1E-02	1.93*	0.15	2.08*
Gallic acid	Benzene and substituted derivatives	2.1E-07	1.7E-05	1.6E-05	6.34*	− 0.09	6.25
Gentisic acid	Benzene and substituted derivatives	2.1E-07	4.4E-05	1.5E-04	7.71*	1.77*	9.48*
Cis-aconitic acid	Carboxylic acids and derivatives	1.8E-02	1.0E-02	1.2E-02	− 0.85*	0.26	− 0.58*
Gamma-glutamyl leucine	Carboxylic acids and derivatives	2.1E-03	5.2E-04	2.9E-04	− 2.01*	− 0.84	− 2.86*
L-Histidine	Carboxylic acids and derivatives	1.1E-04	3.3E-04	3.4E-04	1.58*	0.04	1.63*
L-Methionine	Carboxylic acids and derivatives	1.9E-04	1.6E-02	1.5E-02	6.40*	− 0.09	6.30*
L-Tyrosine	Carboxylic acids and derivatives	1.5E-04	5.6E-04	5.5E-04	1.90*	− 0.03	1.87*
L-Valine	Carboxylic acids and derivatives	1.3E-04	7.8E-04	7.2E-04	2.58*	− 0.12	2.47
Ornithine	Carboxylic acids and derivatives	1.6E-03	1.3E-02	1.6E-02	3.02	0.30*	3.32
Pipecolic acid	Carboxylic acids and derivatives	2.3E-03	3.6E-02	3.5E-02	3.97*	− 0.04	3.93*
Pyroglutamic acid	Carboxylic acids and derivatives	2.1E-07	5.5E-05	4.0E-05	8.03*	− 0.46	7.57
Succinic acid	Carboxylic acids and derivatives	4.1E-05	5.1E-04	5.7E-04	3.64*	0.16	3.80*
Trans-aconitic acid	Carboxylic acids and derivatives	3.5E-04	1.5E-03	1.6E-03	2.10*	0.09	2.19*
3,4-Dihydroxy-trans-cinnamate	Cinnamic acids and derivatives	2.2E-05	9.0E-05	1.3E-04	2.03*	0.53	2.56*
Coumarin	Coumarins and derivatives	1.8E-03	8.0E-03	9.0E-03	2.15*	0.17	2.32*
Apigenin	Flavonoids	1.8E-04	1.4E-03	2.0E-03	2.96*	0.51	3.47*
Chrysoeriol	Flavonoids	3.3E-04	5.5E-03	4.0E-03	4.06*	− 0.46*	3.60*
Isorhamnetin	Flavonoids	7.3E-05	5.7E-04	4.7E-04	2.96*	− 0.28	2.69*
Malvidin 3-glucoside	Flavonoids	3.7E-02	4.1E-03	7.5E-03	− 3.17	0.87	− 2.30*
Petunidin 3-glucoside	Flavonoids	3.5E-03	5.7E-04	1.1E-03	− 2.62*	0.95	− 1.67*
Malic acid	Hydroxy acids and derivatives	3.0E-03	2.7E-04	1.5E-04	− 3.47	− 0.85*	− 4.32
Adenine	Imidazopyrimidines	1.3E-02	1.8E-04	1.1E-04	− 6.17*	− 0.71	− 6.88*
Uric acid	Imidazopyrimidines	2.1E-07	3.1E-05	2.9E-05	7.21*	− 0.10	7.11*
(Â ±)-Tryptophan	Indoles and derivatives	2.7E-02	2.8E-02	3.4E-02	0.05	0.28*	0.33*
1H-Indole-3-carboxaldehyde	Indoles and derivatives	2.6E-05	8.1E-06	6.8E-06	− 1.68*	− 0.25	− 1.93*
Beta-Carboline	Indoles and derivatives	2.2E-05	3.3E-04	1.5E-04	3.91*	− 1.14*	2.77*
Ile Gly	Mini peptide	2.8E-04	2.9E-03	1.7E-03	3.37*	− 0.77*	2.60*
Ser Gly Ile	Mini peptide	8.7E-05	1.7E-04	1.1E-04	0.97*	− 0.63*	0.34
Ser Val Glu	Mini peptide	7.3E-04	1.9E-04	1.1E-04	− 1.94*	− 0.79	− 2.73*
Acetylcholine	Organonitrogen compounds	1.4E-04	3.2E-02	3.4E-02	7.84*	0.09	7.92*
Adenosine	Purine nucleosides	1.7E-01	1.2E-03	1.4E-03	− 7.15	0.22	− 6.92*
Nicotinic acid	Pyridines and derivatives	1.9E-03	2.5E-03	2.5E-03	0.40*	0.00	0.40*
Pyridoxine	Pyridines and derivatives	4.4E-04	1.6E-03	1.7E-03	1.86*	0.09	1.95*
Cytarabine	Pyrimidine nucleosides	8.4E-03	1.3E-03	2.9E-04	− 2.69*	− 2.16*	− 4.86*
Thymidine	Pyrimidine nucleosides	3.1E-04	2.7E-04	7.7E-05	− 0.20	− 1.81*	− 2.01*
*p*-coumaric acid	Hydroxycinnamic acids	2.0E-05	9.2E-06	1.2E-05	− 1.14*	0.34	− 0.79
Caffeic acid	Hydroxycinnamic acids	7.9E-06	6.3E-04	7.3E-04	6.33*	0.21	6.53*
Herniarin	Coumarins and derivatives	1.9E-03	8.1E-03	8.1E-03	2.11*	0.00	2.11*
Trans-cinnamic acid	Cinnamic acids and derivatives	2.6E-03	7.0E-03	6.8E-03	1.41*	− 0.04	1.37*

*FM*, fresh sorghum forage before ensiling; *CON*, sorghum silage without *L. plantarum* inoculation (control); *LP*, sorghum silage with *L. plantarum* inoculation

* Indication of a significant effect (*P* < 0.05)

Irrespective of LP inoculation, ensiling fermentation increased the relative concentration of several metabolites with biofunction, including gallic acid, chrysoeriol, apigenin, caffeic acid, herniarin, trans-cinnamic acid, isorhamnetin, and 3,4-dihydroxy-trans-cinnamate in both low- and high-DM silages. Conversely, *p*-coumaric acid concentration decreased in both low- and high-DM silages during ensiling fermentation.

## Discussion

### Silage fermentation

The appropriate moisture content at ensiling is a crucial factor in determining the quality of silage fermentation (Muck 1990; Guo et al. 2013). However, these effects could be different depending on the forage type and maturity stage. For example, Liu et al. (2011) reported that wilting reduced acetic acid concentration and improved the fermentation quality in stylo (*Stylosanthes guianensis* Swartz) silage. Greater acetic acid concentration may prolong aerobic stability as acetic acid is recognized for its inhibitory effects on yeasts and molds, which are the typical spoilage organisms during aerobic conditions (Muck 2010). Guo et al. (2013) reported a decline in silage fermentation acids (lactate and acetate) in first-cut grass silages as the DM content at ensiling increased from 18 to 49%. Conversely, in second-cut grass silages, the forage DM content had no impact on lactate formation in 60-day silages, but a progressive increase in DM content at ensiling resulted in a decline in silage acetate concentration (after 60 days).

Lower silage pH in LP-inoculated silages was expected as inoculation with homolactic acid bacteria has usually contributed to higher lactic acid production (McDonald et al. 1991). However, the extent of pH reduction in response to homolactic inoculants depends on forage biomass, as higher acidification usually occurs in forages with lower buffering capacity (Muck and Kung 1997). Increased silage pH in high- vs. low-DM silages was likely a function of greater lactic acid formation in low-DM silages. In the first-cut grass silages, Guo et al. (2013) also reported that silage pH increased from 4.83 to 5.10 as the forage DM content at ensiling increased from 18 to 49%. However, an opposite trend was identified in second-cut grass silages.

Although LP inoculation did not have a significant impact on DM loss during ensiling, there was a significant difference in DM loss between low- and high-DM sorghum silages. The low-DM silage experienced a higher DM loss of 14.4% vs. only a 6.62% loss in high-DM silage. The loss of DM in silages is undesirable because it implies the wastage of valuable feed nutrients that could have been utilized by animals (Robinson et al. 2016). The variation in DM loss resulting from different DM contents during ensiling can be attributed to the presence of a highly active microbial population and a faster rate of fermentation in low-DM silage, as these factors may promote oxidation of organic matter (Wilkinson and Davies 2013; Kim et al. 2016). Ensiling low-DM forages may delay pH reduction, possibly causing a less inhibitory effect on the growth and activity of undesirable microorganisms, which thus may increase nutrient loss during the early stages of ensiling fermentation (Ellis et al. 2016; Xia et al. 2023).

### Microbial diversity

The fermentation process during ensiling, LP inoculation, and DM content at ensiling resulted in significant differences in bacterial diversity based on Chao1, an estimator of species richness, and Shannon index, a measure of species diversity (Ogunade et al. 2018; Dong et al. 2020). In support, previous studies have also reported that ensiling fermentation would decrease the richness and diversity of the bacterial community residing within the silage mass (Wang et al. 2020; Xu et al. 2021). Some epiphytic bacteria are unable to adapt to anaerobic conditions, particularly the low pH of silage mass. This may result in their disappearance during ensiling, potentially restricting microbial diversity within the acidic environment of the silage (Méndez-García et al. 2015; Zheng et al. 2017; Guan et al. 2020).

### Dynamic changes in bacterial communities

In support of our finding that epiphytic lactobacilli abundance was very low in both low- or high-DM sorghum, Yan et al. (2019b) and Guan et al. (2020) also identified a very low abundance of epiphytic lactobacilli communities in Italian ryegrass and Napier grass, respectively. Keshri et al. (2018) also reported that *Lactobacillus* species existed at a much lower abundance in fresh corn forage but represented the dominant species in 90-day silages (both non-inoculated and LP-inoculated silages). Domination of LAB in a silage microbial community is crucial for successful silage fermentation, playing a key role in lactic acid production and acidification during the ensiling fermentation in different forages (Kim et al. 2021; Mu et al. 2021). Plant species, climatic conditions, and the type of fertilizer used have been identified as potential factors determining the colonization and, thus, the abundance of epiphytic bacterial communities (McGarvey et al. 2013; McAllister et al. 2018).

Consistent with our finding that *L. buchneri* was the dominant species in 90-day silages, a corn silage study reported that although LP was dominant at the beginning of the silage fermentation, *L. buchneri* began to dominate the silage microbial community after 7 days of fermentation and was the most prevalent species after 60 days of ensiling (Zhou et al. 2016). Xu et al. (2020) also reported that LP inoculation of sainfoin raised the relative abundance of *L. buchneri* while decreasing the abundance of other species after 90 days of silage fermentation. Ferrero et al. (2019) reported that the relative abundance of *L. buchneri* was higher in the stable phase of silage fermentation than in the early stage. In agreement, Weinberg and Chen (2013) reported that as silage fermentation progresses, lactic acid concentration decreases while acetic acid concentration increases in both wheat and corn silage, which was related to the domination of *L. buchneri* within the silage microbial community. *L. buchneri* abundance in silages may suggest the tolerance of this bacterium to the acidic silage environment and its strong competition with other silage microbial communities at the late phase of ensiling fermentation (Guo et al. 2018). Contrary to our observation, Guo et al. (2018) reported that *L. buchneri* represented a small proportion of the microbial community in alfalfa silage when sampled at 60 or 90 days. The inconsistency likely originates from the differences in forage species as well as the epiphytic bacterial composition before ensiling (Parvin and Nishino 2009). Silage microbial community is a critical factor influencing silage quality parameters (Ni et al. 2018; Yang and Wang 2018). In this experiment, silage quality parameters were limited to acidity and organic acids, and additional information is needed to better interpret the differences in silage quality parameters among the silage groups.

### Metabolome profiling

During the ensiling fermentation, proteins may undergo proteolysis by plant proteases, leading to their degradation into peptides and free amino acids (McDonald et al. 1991). A rapid decrease in silage pH may impede proteolysis (Guo et al. 2007). By adding homolactic LAB during ensiling, lactic acid may accumulate faster in the initial stages of fermentation, which aids in lowering the pH at a quicker rate (Ranjit and Kung 2000; Ávila et al. 2014). This may help to explain why fewer small peptides existed in silages inoculated with LP as a faster pH decline during the early phases of ensiling may contribute to faster inhibition of proteolysis dynamics, thereby reducing hydrolysis of forage proteins into peptides, free amino acids, and ammonia (Guo et al. 2007). However, it should be noted that this explanation is speculative as we did not collect the pH data during the early stage of fermentation in this experiment.

More recently, Xia et al. (2023) investigated the impact of *L. rhamnosus* inoculation on the metabolome profile of low-DM (17.7%) Italian ryegrass silage. The authors reported that this homofermentative strain resulted in an upregulation of flavonoid compounds in the flavone and flavonol biosynthesis pathway, which thereby increased apigenin concentration after 60 days of ensiling fermentation. An untargeted metabolomics of *Broussonetia papyrifera* silage demonstrated an increased abundance of apigenin with LP inoculation (Niu et al. 2022). Zou et al. (2023) also reported an accumulation of apigenin as the ensiling fermentation of *Moringa oleifera* leaves progressed. In the animal nutrition context, flavonoids, including apigenin, have shown anti-oxidative and anti-inflammatory properties with potential effects on rumen fermentation function, animal productivity, and health, particularly increasing resilience during stress exposure (Olagaray and Bradford 2019). An in vitro study demonstrated the modifying effect of apigenin on bovine abomasal contractility as they reported the myorelaxation effect of apigenin on smooth muscles in a dose-dependent manner (0.1 to 100 µM; Mendel et al. 2016).

Increased accumulation of caffeic acid with ensiling fermentation could possibly be explained by the action of metabolic enzymes of LP hydrolyzing phenolic acids to derivatives, such as caffeic acid. This observation may help to explain the increased antioxidant activity resulting from LAB fermentation in food and by-products (Khubber et al. 2022). Lactic acid fermentation of apple juice increased caffeic acid concentration, but decreased total phenols, highlighting the bioconversion of phenols to individual phenolic acids (Wu et al. 2020). Caffeic acid may modulate methanogenesis and rumen fermentation by influencing the growth and activity of cellulolytic bacteria residing in the rumen ecosystem. For example, Jin et al. (2021) reported the in vitro anti-methanogenic properties of caffeic acid in a high-forage treatment.

*P*-coumaric acid in plant cell walls is primarily bound to lignin via ester bonds, and it has been suggested that LAB can degrade these ester bonds during ensiling fermentation and release phenolic acids linked by esters (Cao et al. 2016; Xie et al. 2021). LAB metabolism during ensiling fermentation includes dynamic processes, including de-esterification, hydrolysis, or transformation of phenolic compounds (Rodríguez et al. 2009; Khubber et al. 2022). A plausible mechanism contributing to the reduction in the concentration of *p*-coumaric acid in ensiled forages may involve its conversion into other phenolic compounds during the ensiling fermentation process. For example, it has been demonstrated that *L. plantarum* possesses the capability to convert *p*-coumaric acid into vinyl phenol through the action of the phenolic acid decarboxylase enzyme (Rodríguez et al. 2009). In support of our finding, Wang et al. (2022) also reported that during 60 days of ensiling whole-plant corn forage, *p*-coumaric acid concentration decreased, the extent of which was greater in LP-inoculated silage. Giuburuncă et al. (2015) reported that caffeic acid and *p*-coumaric acid addition at a concentration of 6 m*M* resulted in a decrease in ruminal methane emissions without affecting other ruminal fermentation parameters. Adding hydroxycinnamic acids (caffeic acid and *p*-coumaric acid) to animal diets has resulted in improved growth performance, animal health, and meat quality (Jiang and Xiong 2016; Waghorn and McNabb 2003; Peña-Torres et al. 2019).

Accumulation of gallic acid by the ensiling fermentation in both low- and high-DM forages aligns with previous studies that have shown anaerobic fermentation to be conducive to the formation and accumulation of gallic acid (Huang et al. 2016; Zhang et al. 2020). For example, Zhang et al. (2020) reported that gallic acid concentration increased in pickled tea after 18 days of anaerobic fermentation and attributed this biotransformation to a decrease in epiafzelechin-3-O-gallate, epicatechin gallate, and 7-galloylcatechin, which are likely key precursors to gallic acid formation during anaerobic fermentation. Previous studies have reported that gallic acid addition to whole-plant soybean silage (Wang et al. 2021) and high-moisture mulberry leaves (He et al. 2020) improved silage fermentation quality. Using the rumen simulation technique, Wei et al. (2019) reported that methane production decreased after 24 and 48 h when gallic acid was added at 10 mg/g DM, while fiber degradability increased. Additionally, as gallic acid concentration increased (0 to 20 mg gallic acid/g DM), rumen ammonia-N concentration decreased, an indication of improved N-use efficiency.

There is conflicting information in the literature about how the concentration of chrysoeriol metabolite changes during ensiling fermentation. Su et al. (2023) reported that after fermenting whole-plant corn forage (which had a DM content of 25.5%) for 60 days, the chrysoeriol concentration increased. However, Xu et al. (2020) studied the biotransformation of phenolic compounds in sainfoin silage and found that chrysoeriol concentration increased relative to fresh biomass. This suggests that the biotransformation of phenolic compounds during ensiling fermentation depends on the type of substrate, as well as the species and strain dominating lactic acid fermentation.

Isorhamnetin is a flavonoid compound with anti-inflammatory and antioxidant properties (Gong et al. 2020). Gu et al. (2019) reported a higher concentration of isorhamnetin metabolite in *Hippophae rhamnoides* leaves after undergoing a fermentation process and attributed this increase to the deglycosylation reaction facilitated by *β*-glucosidase occurring during this bioconversion process. This explanation was supported by a decrease in flavonoid glycosides after the fermentation. Sun et al. (2021) also reported that LP inoculation of *Cyperus esculentus* L. leaves followed by 60-day ensiling resulted in the accumulation of isorhamnetin. Similarly, Su et al. (2023) reported an increased concentration of isorhamnetin in corn silage after 60 days of fermentation, regardless of LP inoculation.

In conclusion, this study provided a thorough description of the microbiome and metabolome alterations occurring during the ensiling fermentation of whole-plant sorghum. Ensiling sorghum forage with a lower DM content led to higher lactic and acetic acid production and a lower pH in the silage after a 90-day fermentation period. Microbiome analysis suggested that *Pseudomonas congelans* represented the dominant species of the epiphytic microbiota in both DM contents before ensiling, but *L. buchneri* represented the dominant species at the end of ensiling (day 90). Metabolome profiling analysis identified variations in the relative concentration of several metabolites as a function of LP inoculation and DM content at ensiling, highlighting the importance of these factors in promoting the biosynthesis pathways or breakdown of metabolites, which may imply the involvement of microbial processes either through the synthesis pathway, transformation, or increased release during the ensiling fermentation.

## Data Availability

The data that support the findings of this study are available from the corresponding author upon reasonable request. Raw sequencing files and associated metadata have been deposited at NCBI's Sequence Read Archive (accession PRJNA1040454), http://www.ncbi.nlm.nih.gov/sra.
